# Risk Factors Associated With Progression to Surgical Release After Injection of Trigger Digits

**DOI:** 10.5435/JAAOSGlobal-D-20-00159

**Published:** 2021-07-07

**Authors:** H. Paco Kang, Venus Vakhshori, Kurt Mohty, Ali Azad, Rachel Lefebvre

**Affiliations:** From the Department of Orthopaedic Surgery, University of Southern California, Los Angeles, CA.

## Abstract

**Introduction:**

The mainstay of trigger finger treatment is a corticosteroid injection of the affected digits and is associated with a very high success rate. However, some patients do not respond to nonsurgical management and undergo subsequent surgical release. The purpose of this study is to investigate the comorbidities that predispose patients to progressing from injection to surgical release.

**Methods:**

Patient data were obtained from a national insurance database. All patients aged 20 years or older who underwent trigger digit injection were included. Any injection that did not specify the digit was excluded. Subsequent procedures, including repeat injection and surgical release, were identified using relevant Current Procedural Terminology codes. A multivariate model was constructed to evaluate potential risk factors for requiring release after prior injection of the same digit. Stepwise backward selection was used to retain significant variables.

**Results:**

A total of 42,537 trigger digits were identified in 31,830 patients, most of whom were female. The right hand was affected more commonly than the left. The middle and ring fingers were the most commonly affected digits. Over 80% of all trigger digits underwent only a single injection, and approximately 90% of injected digits did not require subsequent release. In the multivariate model, factors associated with higher risk of release were male sex, involvement of additional digits, multiple injections of the same digit, chronic pulmonary disease, HIV/AIDS, obesity, alcohol abuse, and depression. The model also found small fingers to be less likely to progress to release.

**Discussion:**

Patients with the risk factors identified in this study are more likely to progress to surgical release after trigger finger injection. Although prospective studies are required, the information may be beneficial in counseling patients and their treatment options.

Stenosing tenosynovitis of the digits, also known as trigger finger, is a common tendinopathy in the hand, with a recent reported prevalence of approximately 1%.^1^ Normal digit flexion requires the flexor tendons to smoothly glide through a pulley system. This phenomenon is facilitated by the presence of a double synovial sheath within the pulley system. Triggering can occur when inflammation contributes to a size discrepancy of the retinacular sheath and the flexor tendons.^2,3^ The exact etiology of trigger finger is still unknown, although it is thought to be multifactorial.^3^ Presenting symptoms can vary. Although many patients will complain of triggering, clicking, or catching of the involved digit, some patients may only complain of pain at the level of the A1 pulley or report the presence of a painful nodule over the metacarpophalangeal joint.^2-4^

Initial management of trigger finger is often nonsurgical and can involve careful observation alone, activity modification, oral NSAIDs, splinting, corticosteroid injections, or NSAID injections.^3,5,6,7,8^ Some studies advocate for splinting alone as first-line treatment (especially for patients complaining of morning stiffness) with up to 66% of patients needing no further treatment at one year.^3,5^ Corticosteroid injections are also frequently used as first-line nonsurgical therapy due to their ease of use, low risk, and excellent treatment response with complete symptom relief reported in 60% to 90% of patients.^4,9,10^ Open or percutaneous surgical release is a treatment option for patients with continued symptoms despite nonsurgical treatment or for those with a locked digit.

Multiple studies have attempted to identify risk factors associated with progression to surgery, but the literature has somewhat conflicting conclusions. Some studies identify diabetes mellitus (DM), involvement of multiple digits, a history of other tendinopathies of the upper extremity, trigger thumbs, advanced symptoms, symptom duration greater than 6 months, diffuse type stenosing tenosynovitis, triamcinolone injection (compared with dexamethasone), and younger age to be risk factors for trigger finger recurrence.^3,5-7,10,11,12,13,14,15,16^ However, these very same studies have provided contradictory results arguing that symptom severity, duration of symptoms, age, trigger thumbs, and even DM are not risk factors for recurrence.^10,14,17,18^

Although trigger finger is common, there are limited and inconsistent data exploring why certain patients do not respond to nonsurgical management as well as others. The purpose of this retrospective database study is to investigate factors associated with progression to surgery (indicating continuing or recurrent symptoms) after injection of trigger finger.

## Methods

The Humana private insurance database was retrospectively reviewed using PearlDiver software (PearlDiver). This is a commercially available administrative database that consists of approximately 24 million patient records from 2007 to 2016. The database spans all age groups and represents geographic regions throughout the United States. The database allows for the querying of patients by both *International Classification of Diseases* (*ICD*) and Current Procedural Terminology (CPT) codes. All data are deidentified, and therefore, no human subject approval was required.

All patients aged 20 years and older undergoing trigger injection at least once were initially identified for inclusion using CPT codes 20550 and 20551. Subsequent trigger release was identified using CPT code 26055. The digit on which either injections or releases were performed was identified using CPT modifier codes. Exclusion criteria were patients younger than 20 years, to exclude pediatric or congenital trigger fingers, and lack of digit specification.

Each component of the 31-item Elixhauser Comorbidity Index was identified using relevant *ICD-9* and *ICD-10* codes (Table 1).^19^ To identify risk factors associated with the need for future surgical release, a multivariate logistic regression model was constructed using the following independent variables: the presence of any of the Elixhauser Comorbidity Index comorbidities at the time of initial injection, as well as age group, sex, affected digit, multiple digit involvement, and multiple injections of the affected digit. The initial model (Supplemental Table 1, http://links.lww.com/JG9/A136) was subsequently refined using a backward selection procedure using *P* = 0.10 for entry and 0.05 for retention in the final model.^10^ Statistical analysis was performed using the R statistical package that is part of the PearlDiver Software (R Foundation).

**Table 1:** Codes Used for Comorbidity Screening

Comorbidity/Disease	*ICD-9* Codes Used	*ICD-10* Codes Used
Congestive heart failure	398.91, 402.01, 402.11, 402.91, 404.01, 404.03, 404.11, 404.13, 404.91, 404.93, 425.4-9, 428.x	I09.9, I11.0, I13.0, I13.2, I25.5, I42.0, I42.5-I42.9, I43.x, I50.x, P29.0
Cardiac arrhythmias	426.0, 426.13, 426.7, 426.9, 426.10, 426.12, 427.0-4, 427.6-9, 785.0, 996.01, 996.04, V45.0, V53.3	I44.1-3, I45.6, I45.9, I47.x-I49.x, R00.0, R00.1, R00.8, T82.1, Z45.0, Z95.0
Valvular disease	093.2, 394.x-397.x, 424.x, 746.3-746.6, V42.2, V43.3	A52.0, I05.x-I08.x, I09.1, I09.8, I34.x-I39.x, Q23.0-3, Z95.2-4
Pulmonary circulation disorders	415.0, 415.1, 416.x, 417.0, 417.8.417.9	I26.x, I27.x, I28.0, I28.8, I28.9
Peripheral vascular disorders	093.0, 437.3, 440.x, 441.x, 443.1-9, 447.1, 557.1, 557.9, V43.4	I70.x, I71.x, I73.1, I73.8, I73.9, I77.1, I79.0, I79.2, K55.1, K55.8, K55.9, Z95.8, Z95.9
Uncomplicated hypertension	401.x	I10.x
Complicated hypertension	402.x-405.x	I11.x-I13.x, I15.x
Paralysis	334.1, 342.x, 343.x, 344.0-6, 344.9	G04.1, G11.4, G80.1, G80.2, G81.x, G82.x, G83.0-4, G83.9
Other neurologic disorders	331.9, 332.0, 332.1, 333.4, 333.5, 333.92, 334.x-335.x, 336.2, 340.x, 341.x, 345.x, 348.1, 348.3, 780.3, 784.3	G10.x-G13.x, G20.x-G22.x, G25.4, G25.5, G31.2, G31.8, G31.9, G32.x, G35.x-G37.x, G40.x, G41.x, G93.1, G93.4, R47.0, R56.x
Chronic pulmonary disease	416.8, 416.9, 490.x-505.x, 506.4, 508.1, 508.8	I27.8, I27.9, J40.x-J47.x, J60.x-J67.x, J68.4, J70.1, J70.3
Uncomplicated diabetes	250.0-3	E10.0, E10.1, E10.9, E11.0, E11.1, E11.9, E12.0, E12.1, E12.9, E13.0, E13.1, E13.9, E14.0, E14.1, E14.9
Complicated diabetes	250.4-250.9	E10.2-8, E11.2-8, E12.2-8, E13.2-8, E14.2-8
Hypothyroidism	240.9, 243.x, 244.x, 246.1, 246.8	E00.x-E03.x, E89.0
Renal failure	403.01, 403.11, 403.91, 404.02, 404.03, 404.12, 404.13, 404.92, 404.93, 585.x, 586.x, 588.0, V42.0, V45.1, V56.x	I12.0, I13.1, N18.x, N19.x, N25.0, Z49.0, Z49.2, Z94.0, Z99.2
Liver disease	070.22, 070.23, 070.32, 070.33, 070.44, 070.54, 070.6, 070.9, 456.0-456.2, 570.x, 571.x, 572.2-572.8, 573.3, 573.4, 573.8, 573.9, V42.7	B18.x, I85.x, I86.4, I98.2, K70.x, K71.1, K71.3-K71.5, K71.7, K72-K74.x, K76.0, K76.2-K76.9, Z94.4
Peptic ulcer disease, excluding bleeding	531.7, 531.9, 532.7, 532.9, 533.7, 533.9, 534.7, 534.9	K25.7, K25.9, K26.7, K26.9, K27.7, K27.9, K28.7, K28.9
HIV/AIDS	042-044.x	B20-B22.x, B24.x
Lymphoma	200.x, 203.0, 238.6	C81-C85.x, C88.x, C96.x, C90.0, C90.2
Metastatic cancer	196-199.x	C77-C80.x
Solid tumor without metastasis	140-172.x, 174-195.x	C00-C26.x, C30-C34.x, C37-C41.x, C43.x, C45-C58.x, C60-C76.x, C97.x
Rheumatoid arthritis and collagen vascular diseases	446.x, 701.0, 710.0-710.4, 710.8, 710.9, 711.2, 714.x, 719.3, 720.x, 725.x, 728.5, 728.89, 729.30	L94.0, L94.1, L94.3, M05.x, M06.x, M08.x, M12.0, M12.3, M30.x, M31.0- M31.3, M32-M35.x, M45.x, M46.1, M46.8, M46.9
Coagulopathy	286.x, 287.1, 287.3-287.5	D65-D68.x, D69.1, D69.3-D69.6
Obesity	278.0	E66.x
Weight loss	260-263.x, 783.2, 799.4	E40-E46.x, R63.4, R64
Fluid and electrolyte disorders	253.6, 276.x	E22.2, E86.x, E87.x
Blood loss anemia	280.0	D50.0
Deficiency anemia	280.1-280.9, 281.x	D50.8, D50.9, D51-D53.x
Alcohol abuse	265.2, 291.1-291.3, 291.5-291.9, 303.0, 303.9, 305.0, 357.5, 425.5, 535.3, 571.0-571.3, 980.x, V11.3	F10, E52, G62.1, I42.6, K29.2, K70.0, K70.3, K70.9, T51.x, Z50.2, Z71.4, Z72.1
Drug abuse	292.x, 304.x, 305.2-305.9, V65.42	F11-F16.x, F18.x, F19.x, Z71.5, Z72.2
Psychoses	293.8, 295.x, 296.04, 296.14, 296.44, 296.54, 297.x, 298.x	F20.x, F22-F25.x, F28.x, F29.x, F30.2, F31.2, F31.5
Depression	296.2, 296.3, 296.5, 300.4, 309.x, 311	F20.4, F313-F315, F32.x, F33.x, F34.1, F41.2, F43.2

*ICD* = *International Classification of Diseases*

## Results

A total of 42,537 injected trigger digits were identified in 31,830 patients; approximately 62% of whom were female, and 45% of whom were diabetic. Patients aged 65 to 69 years, followed by those aged 70 to 74 years, were the most commonly treated age groups (Table 2). The right hand was affected more often than the left, with the middle and ring fingers being the most commonly affected digits. Additional digits were frequently involved (Table 3). In total, 6849 digits (16.10%) underwent multiple injections of the same digit. Surgical release was not commonly performed, with an overall rate of 10.7% of digits progressing to surgery over time with the small finger being least likely to require surgical release (Table 3).

**Table 2:** Demographic Information

Age (yr)	
20-24	36 (0.11%)
25-29	52 (0.16%)
30-34	133 (0.42%)
35-39	236 (0.74%)
40-44	416 (1.31%)
45-49	799 (2.51%)
50-54	1753 (5.51%)
55-59	2512 (7.89%)
60-64	2955 (9.28%)
65-69	7453 (23.42%)
70-74	6545 (20.56%)
75-79	4425 (13.90%)
80-84	2613 (8.21%)
85-89	990 (3.11%)
90+	912 (2.87%)
Sex	
Female	19674 (61.81%)
Male	12156 (38.19%)
Diabetes	14441 (45.37%)
Total	31830

**Table 3:** Summary of Findings

Digit	Trigger Injections	Involvement of Additional Digits	Multiple Injections of Same Digit	Underwent Release	Did Not Undergo Release
Left					
Thumb	4472	1336 (29.9%)	621 (13.9%)	407 (9.1%)	4065 (90.9%)
Index	2202	1208 (54.9%)	337 (15.3%)	193 (8.8%)	2009 (91.2%)
Middle	6121	2772 (45.3%)	1086 (17.7%)	708 (11.6%)	5413 (88.4%)
Ring	4847	2247 (46.4%)	789 (16.3%)	539 (11.1%)	4308 (88.9%)
Small	1168	641 (54.9%)	134 (11.5%)	75 (6.4%)	1093 (93.6%)
Right					
Thumb	5321	1497 (28.1%)	770 (14.5%)	589 (11.1%)	4732 (88.9%)
Index	2770	1520 (54.9%)	416 (15.0%)	246 (8.9%)	2524 (91.1%)
Middle	7720	3390 (43.9%)	1383 (17.9%)	975 (12.6%)	6745 (87.4%)
Ring	6307	2825 (44.8%)	1108 (17.6%)	709 (11.2%)	5598 (88.8%)
Small	1609	864 (53.7%)	205 (12.7%)	99 (6.2%)	1510 (93.8%)

Over 80% of all trigger digits underwent only a single injection (Table 4). The percentage of digits requiring two injections was 13.2%, which ranged from 10.4% to 14.6% depending on the digit involved (Table 4). The proportion of digits requiring three or more injections was even lower, at 2.9%, which ranged from 1.6% to 3.5% depending on the digit involved (Table 4).

**Table 4:** Injection Count by Digit

Digit	1 Injection	2 Injections	3 or More Injections
Left			
Thumb	3851 (86.1%)	520 (11.6%)	101 (2.3%)
Index	1865 (84.7%)	260 (11.8%)	77 (3.5%)
Middle	5035 (82.3%)	872 (14.2%)	214 (3.5%)
Ring	4058 (83.7%)	632 (13.0%)	157 (3.2%)
Small	1034 (88.5%)	121 (10.4%)	13 (1.1%)
Right			
Thumb	4551 (85.5%)	656 (12.3%)	114 (2.1%)
Index	2354 (85.0%)	331 (12.0%)	85 (3.1%)
Middle	6337 (82.1%)	1125 (14.6%)	258 (3.3%)
Ring	5199 (82.4%)	902 (14.3%)	206 (3.3%)
Small	1404 (87.3%)	180 (11.2%)	25 (1.6%)

Several risk factors are significantly associated with different likelihood of surgical trigger digit release. Patients with multiple digits involved and multiple injections of the affected digit were more likely to ultimately undergo surgical release (odds ratio [OR] 1.46 and OR 1.64, respectively, *P* < 0.001). Triggering of the small finger was associated with a lower likelihood of progressing to surgery after injection compared with the rest of the digits (OR 0.92, *P* = 0.03, Table 5). The presence of chronic pulmonary disease (OR 1.10, *P* < 0.001), HIV/AIDS (OR 2.21, *P* < 0.001), obesity (OR 1.17, *P* < 0.001), alcohol abuse (OR 1.65, *P* < 0.001), and depression (OR 1.13, *P* < 0.001) showed an increased association with surgical release, whereas pulmonary circulation disorders (OR 0.83, *P* < 0.001), peripheral vascular disorders (OR 0.85, *P* < 0.001), lymphoma (OR 0.78, *P* < 0.017), and fluid and electrolyte disorders (OR 0.83, *P* < 0.001) showed a decreased association with surgical release (Table 5).

**Table 5:** Multivariate Model Demonstrating the Risk Factors Associated With Surgical Release of a Trigger Digit

Parameter	OR (95% CI)	*P*
Male sex	0.92 (0.88-0.97)	0.0026
Involvement of additional digits	1.46 (1.36-1.56)	<0.0010
Multiple injections of affected digit	1.64 (1.56-1.73)	<0.0010
Digit involved		
Thumb	1.20 (1.12-1.27)	<0.0010
Index	1.19 (1.12-1.27)	<0.0010
Middle	1.41 (1.33-1.50)	<0.0010
Ring	1.21 (1.14-1.29)	<0.0010
Small	0.92 (0.85-0.99)	0.026
Pulmonary circulation disorders	0.83 (0.75-0.91)	<0.0010
Peripheral vascular disorders	0.85 (0.80-0.90)	<0.0010
Chronic pulmonary disease	1.10 (1.05-1.16)	<0.0010
HIV/AIDS	2.21 (1.56-3.13)	<0.0010
Lymphoma	0.78 (0.63-0.95)	0.017
Obesity	1.17 (1.10-1.25)	<0.0010
Fluid and electrolyte disorders	0.83 (0.78-0.88)	<0.0010
Alcohol abuse	1.65 (1.33-2.04)	<0.0010
Depression	1.13 (1.07-1.19)	<0.0010

95% CI = 95% confidence interval, OR = odds ratio

## Discussion

Previous studies evaluating persistent trigger digit after injection have demonstrated a success rate of 40% to 90%.^12,14,20,21,22,23,24,25^ Our results regarding the rate of surgical trigger finger release after injection are consistent with these previously published studies, with slightly above 10% of the injected digits progressing to surgical release. The small finger injection was associated with a lower rate of future surgery, in line with other studies reporting ring, index, and middle fingers as independent predictors for trigger recurrence.^14,15^

This is the largest study to date evaluating risk factors and protective factors associated with progression to trigger finger release surgery after injection. A major finding of our study, in contrast to other published studies, is that neither complicated nor uncomplicated diabetes is significantly associated with requiring repeat injection or surgical release. Diabetes, particularly insulin dependent diabetes mellitus (IDDM), is frequently discussed as a risk factor for both increased rate of trigger finger and decreased rates of successful treatment with corticosteroid injection.^10,13,20,22,26,27^ In a landmark, prospective study, IDDM was associated with lack of response to injection alone in 124 trigger digits in 119 patients.^10^ As the authors note in their article, the study was limited by a small number of patients with IDDM and a low number of the studied outcome, both of which increased the chance of a false-positive error. Another small, well-designed prospective investigation found that the 30 patients with diabetes enrolled in their study were more likely to undergo surgical release when compared with controls.^11^ Perhaps the difference between our data and theirs lies in the severity of the disease: in their study, patients with diabetes seemed to have predominantly advanced disease with high rates of diabetic sequelae including nephropathy, retinopathy, and neuropathy.

Our study is not the first to question the link between DM and trigger finger recurrence after injection. A recent, large investigation evaluated 366 trigger digits at 5 years of follow-up after a single corticosteroid injection.^17^ The authors of this study found that diabetes was not a predictor for treatment success. Similarly, a retrospective study of 286 patients did not find diabetes was a predictor of recurrence after corticosteroid injection.^14^

In these studies and in ours, the large sample size may offer the opportunity to parse out diabetes from associated characteristics that may be confounders associated with true predictors of progression to surgical treatment. DM has been associated with multiple digit involvement.^13^ Triggering of multiple digits has been demonstrated by several studies to be a risk factor for lack of relief from nonsurgical treatment.^7,10,16,18,28^ Specifically, patients with multiple digit involvement have been shown to be 2.4 times more likely to have repeat injection.^10^ Although the effect in our patient population is slightly smaller (OR 1.64), our results support the conclusion that multiple digit involvement, not necessarily DM itself, is associated with surgical release after nonsurgical treatment. The number of times a given triggering digit was injected in patients with diabetes is presented in Supplemental Table 2, http://links.lww.com/JG9/A136.

Our analysis found that patients with pulmonary circulation disorders, peripheral vascular disorders, lymphoma, and fluid and electrolyte disorders were unlikely to undergo release after trigger injection. These factors have not been previously studied. One explanation is a selection bias: patients with these comorbidities are poor surgical candidates^29-31^ leading the patient or clinician to recommend against surgery. However, in our investigation of other serious comorbidities that similarly predict poor surgical outcomes (including liver disease,^32^ anemia,^33^ and renal failure,^34^), these were not associated with a statistically significant difference in rate of surgical management. The underlying reason for these differences between serious comorbidities is not readily apparent and will require additional study.

Another novel finding of our analysis was the high rate of progression to surgery after injection associated with alcohol abuse and HIV/AIDS. HIV is now well known in the medical literature to be associated with a range of inflammatory and rheumatologic disorders,^35^ and although a risk of trigger finger has not been reported, tenosynovitis has been theorized to be a possible adverse effect of antiretroviral treatment and the subsequent immune reconstitution syndrome.^36^ The correlation between alcoholism and trigger finger has not yet been well explored in the literature. However, it has been demonstrated that large quantities of alcohol intake or alcohol dependence lead to an increase in inflammation^37-39^ through oxidative stress, among other mechanisms. This effect may be the basis of our study's findings and represents an opportunity for additional preclinical and clinical studies in this population.

Last, we found that depression was a predictor of requiring future surgery after trigger injection. Although depression has been linked to pain with injection,^40^ this is the first study reporting a link between depression and progression to surgical treatment. Depression severity has previously been correlated with self-reported pain in trigger finger patients, as well as self-reported upper extremity function.^40-42^ Because one of the reasons to perform a release after injection is a persistence of symptoms, it is possible that patients with depression are more likely be dissatisfied with the injection outcomes. Although this may lead these patients and clinicians to surgery, they may not have the same postoperative outcomes as it has been reported that psychosocial factors, and in particular depression, have a large impact on pain and disability on outpatient hand surgery, including trigger release.^42^

The strength of our study is that in comparison to most existing studies examining the progression to surgical release in trigger finger care, it is able to include longitudinal care of the affected patients regardless of clinician or facility. Many studies on this topic are published by hand specialists, often in tertiary referral centers. This database study avoids that inherent bias by capturing the significant number of patients treated by nonsurgical clinicians.^43^ Moreover, our database continues to follow these patients longitudinally, even if they are referred to a different clinician or to a surgeon.

This study is limited by the quality of procedure coding, as with all database studies. Another limitation is that treatment received outside the insurance carrier was not captured, and therefore, patients who switched insurance carriers after the index injection may be misconstrued as having had treatment success. Functional clinical variables such as preoperative symptom severity and duration, hand dominance, treatments before the data collection period, and postoperative hand function could not be obtained in this study. It must also be stressed that the nature of a database study allows us to determine only correlative associations, rather than causation. Similarly, while the large sample size in the study is generally a strength, this can result in statistical significance being found in otherwise clinically unimportant differences. Several of the factors identified, such as depression, have a relatively small effect size as measured by odds ratios, although the *P* value was under the defined threshold.

In conclusion, our results suggest that nearly 90% of trigger digits may be successfully treated with steroid injections, whereas just over 10% of patients do not sufficiently respond to treatment with injections and undergo surgical release. Females, patients with multiple digit involvement, and patients requiring multiple injections were more likely to require surgical release. Medical comorbidities associated with increased odds of progression to surgery after injection include chronic pulmonary disease, HIV/AIDS, obesity, alcohol abuse, and depression. HIV/AIDS and alcohol abuse in particular demonstrated the highest odds for future release after injection, and although additional study is clearly needed on these risk factors, the data presented here can guide expectation management and treatment counseling. On the other hand, involvement of the small finger may be predictive of better response to nonsurgical treatment. The multivariate model did not find diabetes to be a risk factor for progression to surgical care. Although injection can be the definitive treatment for many, identifying risk factors for patients who require multiple injections or surgical release can help guide patient care and expectations.

## Supplementary Material

SUPPLEMENTARY MATERIAL
