# Supplementary material for: Risk Factors Associated With Progression to Surgical Release After Injection of Trigger Digits
Source: J Am Acad Orthop Surg Glob Res Rev. 2021 Jul 7;5(7):e20.00159. doi: 10.5435/JAAOSGlobal-D-20-00159 (PMC8265869; doi:10.5435/JAAOSGlobal-D-20-00159)
Supplement: SUPPLEMENTARY MATERIAL [file jagrr-5-e20.00159-s001.docx]

**Supplemental Table 1: Initial Multivariate Model Demonstrating The Risk Factors Associated With Surgical Release Of A Trigger Digit.**

| **Parameter** | **OR (95%CI)** | **p** |
| --- | --- | --- |
| Male Gender | 0.93 (0.88-0.99) | 0.013 |
| Involvement of Additional Digits | 1.48 (1.38-1.59) | <0.0010 |
| Multiple Injections of Affected Digit | 1.64 (1.55-1.72) | <0.0010 |
| Digit Involved: | 1.15 (1.08-1.23) | <0.0010 |
| Thumb | 1.20 (1.13-1.28) | <0.0010 |
| Index | 1.41 (1.33-1.50) | <0.0010 |
| Middle | 1.22 (1.15-1.30) | <0.0010 |
| Ring | 0.91 (0.84-0.98) | 0.010 |
| CHF | 0.96 (0.89-1.03) | 0.26 |
| Arrhythmia | 1.08 (1.02-1.14) | 0.011 |
| Valvular Disease | 0.99 (0.93-1.06) | 0.79 |
| Pulmonary Circulation Disorders | 0.89 (0.81-0.98) | 0.020 |
| Peripheral Vascular Disorders | 0.93 (0.88-0.99) | 0.026 |
| Simple HTN | 1.11 (1.03-1.19) | 0.006 |
| Complex HTN | 0.97 (0.90-1.05) | 0.46 |
| Paralysis | 1.09 (0.94-1.27) | 0.24 |
| Other Neurological Disorders | 1.05 (0.96-1.14) | 0.27 |
| Chronic Pulmonary Disease | 1.11 (1.05-1.17) | <0.0010 |
| Simple DM | 0.93 (0.87-0.99) | 0.036 |
| Complex DM | 1.00 (0.93-1.08) | 0.94 |
| Hypothyroidism | 1.04 (0.99-1.10) | 0.11 |
| Renal Failure | 0.99 (0.92-1.07) | 0.82 |
| Liver Disease | 0.96 (0.90-1.03) | 0.22 |
| Peptic Ulcer Disease | 1.02 (0.82-1.26) | 0.85 |
| HIV/AIDS | 2.08 (1.46-2.93) | <0.0010 |
| Lymphoma | 0.83 (0.67-1.03) | 0.092 |
| Metastatic Disease | 0.90 (0.78-1.03) | 0.13 |
| Solid Tumors | 1.00 (0.93-1.07) | 0.93 |
| Rheumatoid Arthritis | 0.99 (0.93-1.05) | 0.66 |
| Coagulation Disorders | 0.95 (0.87-1.04) | 0.28 |
| Obesity | 1.11 (1.04-1.18) | 0.0012 |
| Weight Loss | 1.02 (0.91-1.13) | 0.78 |
| Electrolyte Disorders | 0.86 (0.81-0.92) | <0.0010 |
| Blood Loss | 1.04 (0.91-1.17) | 0.57 |
| Anemia | 1.05 (0.98-1.12) | 0.19 |
| Alcohol Abuse | 1.54 (1.24-1.91) | <0.0010 |
| Drug Abuse | 1.04 (0.96-1.14) | 0.33 |
| Psychoses | 1.05 (0.92-1.19) | 0.47 |
| Depression | 1.07 (1.01-1.13) | 0.019 |

OR: Odds ratio; odds ratio of 1 means no difference between groups

95%CI: 95% Confidence interval

**Supplemental Table 2: Injection Count by Digit in Diabetic Patients**

|  | **1 Injection** | **2 Injections** | **3 or More Injections** |
| --- | --- | --- | --- |
| **Left** |  |  |  |
| Thumb | 1609 (85.8%) | 222 (11.8%) | 45 (2.4%) |
| Index | 1011 (84.5%) | 147 (12.3%) | 38 (3.2%) |
| Middle | 2499 (82.3%) | 419 (13.8%) | 120 (4.0%) |
| Ring | 1997 (84.5%) | 291 (12.3%) | 75 (3.2%) |
| Small | 490 (88.9%) | 52 (9.4%) | 9 (1.6%) |
|  |  |  |  |
| **Right** |  |  |  |
| Thumb | 1860 (87.1%) | 236 (11.0%) | 39 (1.8%) |
| Index | 1194 (84.9%) | 172 (12.2%) | 41 (2.9%) |
| Middle | 2941 (82.6%) | 493 (13.8%) | 128 (3.6%) |
| Ring | 2436 (82.4%) | 428 (14.5%) | 91 (3.1%) |
| Small | 658 (87.4%) | 87 (11.6%) | 8 (1.1%) |
